# Therapeutic Effects of Traditional Chinese Medicine on Cardiovascular Diseases: the Central Role of Calcium Signaling

**DOI:** 10.3389/fphar.2021.682273

**Published:** 2021-07-09

**Authors:** Yuxin Li, Zhang Zhang, Sen Li, Tingting Yu, Zhaoqi Jia

**Affiliations:** School of Life Sciences, Beijing University of Chinese Medicine, Beijing, China

**Keywords:** traditional Chinese medicine, cardiovascular diseases, calcium signaling, cardiomyocytes, endothelial cells, vascular smooth muscle cells

## Abstract

Calcium, as a second messenger, plays an important role in the pathogenesis of cardiovascular diseases (CVDs). The malfunction of calcium signaling in endothelial cells and vascular smooth muscle cells promotes hypertension. In cardiomyocytes, calcium overload induces apoptosis, leading to myocardial infarction and arrhythmias. Moreover, the calcium–calcineurin–nuclear factor of activated T cells (NFAT) pathway is essential for expressing the cardiac pro-hypertrophic gene. Heart failure is also characterized by reduced calcium transient amplitude and enhanced sarcoplasmic reticulum (SR) calcium leakage. Traditional Chinese medicine (TCM) has been used to treat CVDs for thousands of years in China. Because of its multicomponent and multitarget characteristics, TCM's unique advantages in CVD treatment are closely related to the modulation of multiple calcium handling proteins and calcium signaling pathways in different types of cells involved in distinct CVDs. Thus, we systematically review the diverse mechanisms of TCM in regulating calcium pathways to treat various types of CVDs, ranging from hypertrophic cardiomyopathy to diabetic heart disease.

## Introduction

Cardiovascular diseases (CVDs) have become common diseases and the main cause of death worldwide (Roth et al., 2017). In 2016, 56 million people died globally, and CVDs caused one-third of the deaths. It is also estimated that 23.6 million people will die from CVDs by 2030 (World Health Organization). The number of patients with CVDs in China is expanding rapidly, reaching more than 290 million, and CVDs are currently the leading cause of mortality (∼40%) (Hu Shengshou et al., 2019). With the increasing number of CVD patients, the cost of CVD treatment has also increased, prioritizing the prevention and treatment of CVDs (Tarride et al., 2009; Bansilal et al., 2015).

Calcium ions (Ca^2+^), as a second messenger, play an important role in different types of cells involved in various CVD conditions, such as hypertension, arrhythmias, and myocardial infarction (Firth et al., 2013; Xia et al., 2016; Mustroph et al., 2017; Wilson et al., 2019). Under physiological conditions, regulation of intracellular Ca^2+^ concentration ([Ca_2+_]i) can effectively change the activity of vasodilation/contraction in vascular smooth muscle cells (VSMCs) (Marchand et al., 2012; Hao et al., 2019). Endothelial cells participate in the modulation of vascular tone by releasing vasoactive agents, such as nitric oxide (NO), which in turn alters [Ca^2+^]_i_ and maintains blood vessel homeostasis (Takeuchi et al., 2004; Filippini et al., 2019). In cardiomyocytes, calcium influx through voltage-dependent calcium channels (VDCCs) activates ryanodine receptors (RyRs) to release calcium from the sarcoplasmic reticulum (SR), which is called calcium-induced calcium release (CICR), and elevated calcium levels activate myofilaments to cause contraction (Bers, 2008; Louch et al., 2015; Han et al., 2019). Dysregulation of Ca^2+^ signaling in the heart is tightly correlated with pathogenesis of CVDs. For example, Ca^2+^ release from nuclear envelope activates CaMKII and promotes nuclear translocation of histone deacetylase 5 (HDAC5), initiating the cardiac hypertrophy (Nakayama et al., 2010). For an inherited arrhythmia called catecholaminergic polymorphic ventricular tachycardia (CPVT), RyRs mutation and β-adrenergic stimulation make SR Ca^2+^ content exceed a threshold, promoting the generation of calcium waves that lead to arrhythmias (Eisner, 2014). Furthermore, Ca^2+^ overload induces apoptosis in cardiomyocytes under pathological conditions, leading to myocardial infarction (Vassalle and Lin, 2004; Jiao et al., 2019), and increased SR Ca^2+^ leakage and reduced SR Ca^2+^ uptake can be normally observed during heart failure (HF) (Kalyanasundaram et al., 2013). Moreover, hypertension is related to the malfunction of calcium signaling in endothelial cells and VSMCs (Touyz et al., 2018; Wilson et al., 2019). Thus, calcium signaling is crucial during the pathogenesis of CVDs, and more detailed mechanisms of CVDs pathogenesis due to Ca^2+^ signaling malfunction are referred to other reviews (Cartwright et al., 2011; Eisner, 2014; Landstrom et al., 2017).

Traditional Chinese medicine (TCM) has been used to treat CVDs for thousands of years, with antioxidative, anti-inflammatory, and protective effects on vascular endothelial cells (Pan et al., 2011; Tao et al., 2012; Liu et al., 2015; Lam et al., 2016). For example, a multicenter clinical trial has revealed that Qiliqiangxin (QLQX) capsules, in addition to standard treatment, effectively decreases the levels of N-terminal pro-B-type natriuretic peptide in patients with chronic heart failure (Li et al., 2013). Furthermore, the clinical intravenous use of TCM for patients with acute myocardial infarction is increasing in China, and *Salvia miltiorrhiza* represents the most common prescription (Spatz et al., 2018). A meta-analysis including seventeen randomized controlled trials suggest the primary end points of patients with unstable angina are reduced by Panax notoginseng saponins, an important effective constituent in *Panax notoginseng* (Duan et al., 2018). Accumulating evidence suggests that calcium signaling plays a central role in the underlying mechanisms of TCM's therapeutic effects (Qi et al., 2008; Wang et al., 2012; Li et al., 2018a; Jiayi et al., 2019; Li et al., 2020). For instance, baicalein, the active ingredient of *Scutellaria baicalensis*, has been shown to reduce calcium levels in cardiomyocytes and prevent heart remodeling (Zhao et al., 2016). Ginseng extract has been reported to reduce the blood pressure of patients in clinical trials, which is beneficial for CVD prevention, and targeting calcium channels in vascular tissues may be one of the underlying mechanisms (Liu and Huang, 2016). Based on the current knowledge, we systematically review the diverse mechanisms of TCM in regulating calcium pathways to treat various types of CVDs.

## Protective Effects of Traditional Chinese Medicine and Its Bioactive Components on Various Types of Cardiovascular Diseases

### Hypertrophic Cardiomyopathy

Several risk factors such as hypertension can induce compensatory muscle tissue thickening and chamber enlargement in the heart, which is called cardiac hypertrophy (Drazner, 2011). For physiological hypertrophy, heart growth or hypertrophy (such as increased heart function caused by regular exercise), is reversible and harmless with no abnormality in the heart structure (Maillet et al., 2013). Pathological hypertrophy, in contrast to physiological hypertrophy, is often linked with interstitial fibrosis and dysregulation of cardiac function, thereby promoting heart failure (HF) and sudden death (Iemitsu et al., 2001; Bernardo et al., 2010; Aoyagi and Matsui, 2011). The characteristics of cardiac pathological hypertrophy at the cellular level include cell enlargement, protein synthesis enhancement, and fetal gene activation (Cox and Marsh, 2014; Tham et al., 2015). Multiple calcium-related signaling pathways are involved in cardiac hypertrophy (Mukherjee and Spinale, 1998; Wilkins and Molkentin, 2004). For example, after binding with Ca^2+^/calmodulin (CaM), calcineurin dephosphorylates nuclear factor of activated T cells (NFAT) in the cytosol and promotes NFAT translocation into the cell nucleus, which regulates gene expression during cardiac hypertrophy (Dewenter et al., 2017).

**FIGURE 1:**
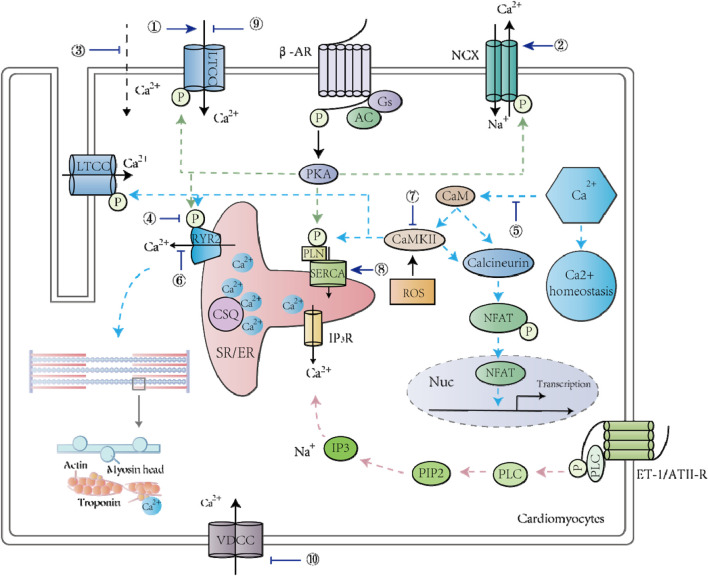
TCM and its active ingredients treat CVDs by regulating calcium signaling pathways in cardiomyocytes. ①Matrine; ②Matrine; ③Changrolin, Paeoniflorin monomer, Dehydroevodiamine alkaloid; ④Stachydrine; ⑤Scutellarin, Astragali Radix; ⑥Senkyunolide A, Smilax glabra flavonoids, Baicalein; ⑦Astragalus polysaccharide; ⑧Baicalein; ⑨6-Gin; Astragaloside IV; Wenxin Keli; Qiliqiangxin; Total flavones from AS; ⑩Senkyunolide A; Ligustrazine.

TCM and its active ingredients can treat myocardial hypertrophy through calcium signaling pathways (Table 1). For instance, Dracocephalum heterophyllum Benth flavonoid (DHBF) has been reported to alleviate myocardial hypertrophy induced by angiotensin II (Ang II), reflected by downregulation of cardiac hypertrophy markers and reduction of cell surface area. DHBF protected hypertrophic cardiomyocytes, presumably by blocking Ang II type 1 receptor and L-type calcium channels (LTCCs) to assist NO release and to reduce intracellular calcium levels (Jiang et al., 2018). Stachydrine (STA), the main biologically active ingredient of *Leonurus heterophyllus*, attenuated cardiac hypertrophy and protected the heart by reducing the amplitude of calcium transients and prolonging the decay constant of calcium transients. The underlying mechanism is to reduce cAMP levels, to inhibit cAMP-dependent protein kinase (PKA) activation, and to prevent phospholamban (PLN) phosphorylation (Zhang et al., 2014). Tanshinone IIA, a compound from the root of *Salvia miltirrhiza*, has been shown to alleviate isoprenaline (ISO)-induced cardiomyocyte hypertrophy because it could attenuate the ISO-mediated upregulation of atrial natriuretic peptide (ANP), brain natriuretic peptide (BNP), calcineurin, NFATc3, and β-myosin heavy chain (β-MHC) (Tan et al., 2011). *Astragalus membranaceus* contains an active component called Astragalus polysaccharide (APS), which has been shown to play an essential role in anti-myocardial hypertrophy by targeting the Ca^2+^-mediated signaling cascade, including prevention of hypertrophy-related NFATc3 nuclear translocation and downregulation of calmodulin-dependent protein kinase II (CaMKII) activity (Dai et al., 2014). Smilax glabra flavonoids (SGF) suppress calcium release from SR by blocking RyR2, thereby protecting myocardial cells from hypertrophy (Shou et al., 2013). Moreover, Cai et al. found that Tu-fu-ling flavonoids (TFLF) could reverse the induction of myocardial hypertrophy by Ang II, and its mechanism might be similarly related to RyR2 and junctophilin-2 (JP2) (Cai et al., 2015). Scutellarin is a bioactive component of *Erigeron breviscapus*, which is widely used in the treatment of CVDs. The cardiac hypertrophy model based on transverse aortic ligation has been applied to study its cardioprotective effect, and the results revealed that scutellarin was capable of treating hypertension and myocardial hypertrophy by modulating the Ca^2+^-mediated calcineurin pathway (Pan et al., 2010). Taken together, TCM could reduce [Ca^2+^]_i_ by blocking either calcium entry through LTCC or calcium release from SR, thereby inhibiting the calcium–calcineurin–NFAT signaling pathway and preventing cardiac hypertrophy (Figure 1).

**TABLE 1:** The mechanism of TCM in the treatment of cardiac hypertrophy through calcium signaling.

Type of TCM	TCM	Type of study	Type of cell	Mechanism of action	References
The bioactive ingredients of TCM	Dracocephalum heterophyllum Benth flavonoid	*In vitro*	Cardiomyocytes	Downregulation of cardiac hypertrophy genes and reducing cell surface area	Jiang et al. (2018)
	Stachydrine	*In vitro*	Ventricular myocytes	Reducing cAMP levels, inhibiting PKA activation and PLN phosphorylation	Zhang et al. (2014)
	Tanshinone IIA	*In vitro*	Ventricular myocytes	Preventing the augment of intracellular calcium transient and inhibiting calcium-mediated calcineurin/NFATc3 pathways	Tan et al. (2011)
	Tu-fu-ling flavonoids	*In vitro*	Cardiomyocytes	Attenuating hypertrophy by restoring the JP2 and RyR2 expressions of cardiomyocytes	Cai et al. (2015)
	Smilax glabra flavonoids	*In vitro*	H9C2	Inhibiting intracellular calcium release	Shou et al. (2013)
	Scutellarin	*In vivo, in vitro*	Cardiomyocytes	Alleviating the increment of free intracellular calcium	Pan et al. (2010)

### Arrhythmia

Arrhythmia is a common heart disease characterized by abnormal heart rhythm, which can be either too fast, i.e., tachycardia, such as atrial fibrillation (AF), or too slow, i.e., bradycardia, which could occur in sick sinus node syndrome (Wang et al., 2017a; Zaza et al., 2018). There are many predisposing factors for arrhythmia. For example, hypoxia and oxidative stress mediate Ca^2+^ overload in myocardial cells and generate heart rhythm irregularities and other myocardial dysfunctions (Guan et al., 2015).

Several studies have evaluated and explored the therapeutic effects of common Chinese medicines and their active ingredients on arrhythmia through modulating cardiac calcium handling (Liu et al., 2017a; Yang et al., 2020a; Tian et al., 2018) (Table 2). The antiarrhythmic effect of matrine in *Sophora tonkinensis* was revealed in a rat arrhythmia model induced by coronary artery ligation. Matrine ameliorated ischemia-mediated abnormal reduction of intracellular Ca^2+^, presumably by enhancing the activity of LTCCs (Li et al., 2009). These results collectively indicated that long-term use of matrine resisted arrhythmia and myocardial infarction and protected heart function (Li et al., 2009). Changrolin, an active component of *Dichroa febrifuga*, could protect against arrhythmia because of its role in inhibiting multiple ion currents, such as the sodium current (*I*_Na_) and calcium currents (*I*_Ca_), and changing the electromechanical properties of isolated rat cardiomyocytes (Chen et al., 2010). As examined by the whole cell patch-clamp technique, paeoniflorin protected against arrhythmias by inhibiting L-type calcium current (*I*_CaL_) to reduce oscillatory afterpotentials or extrasystoles (Wang et al., 2011). Gan et al. found that isorhynchophylline, an alkaloid from *Uncaria rhynchophylla*, had various effects in treating hypertension, arrhythmia, and other diseases (Gan et al., 2011). More specifically, isorhynchophylline alleviated arrhythmia in rats and guinea pigs by inhibiting the calcium current and reducing the action potential duration in cardiomyocytes, leading to a decreased rate of arrhythmia occurrence and mortality (Gan et al., 2011).

**TABLE 2:** The mechanism of TCM in the treatment of arrhythmia through calcium signaling.

Type of TCM	TCM	Type of study	Type of cell	Mechanism of action	References
The bioactive ingredients of TCM	Matrine	*In vivo*, *in vitro*	Cardiomyocytes	Restoring the Ito, recovering the IK1 and the amplitude of [Ca^2+^]_i_	Li et al. (2009)
	Changrolin	*In vitro*	Cardiomyocytes	Blocking sodium and calcium channels	Chen et al. (2010)
	Paeoniflorin monomer	*In vitro*	Cardiomyocytes	Blocking *I*_CaL_, suppression of oscillatory afterpotentials or extrasystoles	Wang et al. (2011)
	Isorhynchophylline	*In vivo*, *in vitro*	Cardiomyocytes	Decreasing action potential duration and inhibiting calcium currents	Gan et al. (2011)
Chinese patent medicine	Wenxin keli	*In vitro*	Cardiomyocytes	Reducing calcium overload	Luo et al. (2017)
	Shensong Yangxin	*In vitro*	Cardiomyocytes	Blocking multiple ion channels	Li et al. (2007)
	Qiliqiangxin	*In vitro*	Ventricular myocytes	Blocking *I*_CaL_	Wei et al. (2012)
				

One of the critical targets of numerous Chinese patent medicines in treating arrhythmia is calcium signaling. For example, Wenxin Keli (WXKL), a widely used TCM in China, prepared from five ingredients: *Polygonatum sibiricum*, *Codonopsis*, *Notoginseng*, amber, *Nardostachys jatamansi* DC. WXKL improved calcium homeostasis by modulating the LTCC, late sodium current (*I*_NaL_), transient outward potassium current (*I*_to_), and the downstream Ca^2+^/CaMKII pathway (Wang et al., 2017a; Xing et al., 2013; Wang et al., 2013; Chen et al., 2013; Xue et al., 2013). Ischemia and reperfusion injury, as modeled by hypoxia and reoxygenation at the cellular level, induce arrhythmia and cell death, which is mainly due to Ca^2+^ overload (Luo et al., 2017). WXKL prevented dysregulation of the *I*_NaL_, sodium-calcium exchanger current (*I*_NCX_) and Ca^2+^ overload induced by ischemia-reperfusion and protected cardiomyocytes from arrhythmia and death (Luo et al., 2017). Some clinical studies have found that WXKL can reduce heart rate, improve heart failure, and relieve heart failure complications (Zheng et al., 2018; Chen et al., 2014). Shensong Yangxin (SSYX) is composed of extracts from 12 herbs, including *Panax ginseng*, *Ophiopogon japonicus*, *Cornus officinalis*, etc. The whole cell patch-clamp technique has been used to examine the cardiac protection mechanism of SSYX, and the results suggested that SSYX alleviated arrhythmia by inhibiting various ion channels (*I*_Ca_, *I*_NaL_, *I*_k_, *I*_to_, and *I*_K1_) (Li et al., 2007). QLQX capsule is composed of 11 herbal medicines such as *Astragali Radix*, *Panax ginseng*, *Alismatis Rhizoma*, and *Carthami Flos*. QLQX capsule has been used in the clinical treatment of arrhythmia (Wei et al., 2012), and experiments in a rabbit model of electrical stimulation-induced AF showed that QLQX effectively rescued the pacing-mediated downregulation of the L-type dihydropyridine receptor (DHPR) and reduced AF inducibility (Tingting et al., 2019). In addition, QLQX effectively blocked *I*_CaL_ in ventricular cardiomyocytes isolated from rats and modulated cardiac function (Wei et al., 2012). Thus, TCM systematically regulated multiple ion currents such as *I*_CaL_ to prevent Ca^2+^ overload and to modulate the electrophysiological properties of cardiomyocytes, which is beneficial for arrhythmia treatment (Figure 1). Indeed, clinical trials showed that western medication plus QLQX reduced the levels of NT-proBNP and could improve the quality of life of patients with heart failure (Li et al., 2013; Wang et al., 2017b).

### Hypertensive Heart Disease

Hypertension is a multifactorial disease and a known risk factor for CVDs (Kokubo and Matsumoto, 2016). The morbidity, mortality, and economic burden attributable to hypertension are high (Bernardes et al., 2013). Hypertension causes blood vessels to become too tight, resulting in atherosclerosis (Zaheer et al., 2016). With plaque formation and blood vessel narrowing, blood flow to the heart muscle will be reduced or interrupted, depriving the heart muscle of oxygen and nutrition, leading to a series of diseases, such as myocardial ischemia and myocardial infarction, coronary heart disease, and myocardial hypertrophy (Bentzon et al., 2014). At present, the pathogenesis of hypertension is mainly related to vascular endothelium malfunction (Spieker et al., 2006; Dharmashankar and Widlansky, 2010).

**FIGURE 2:**
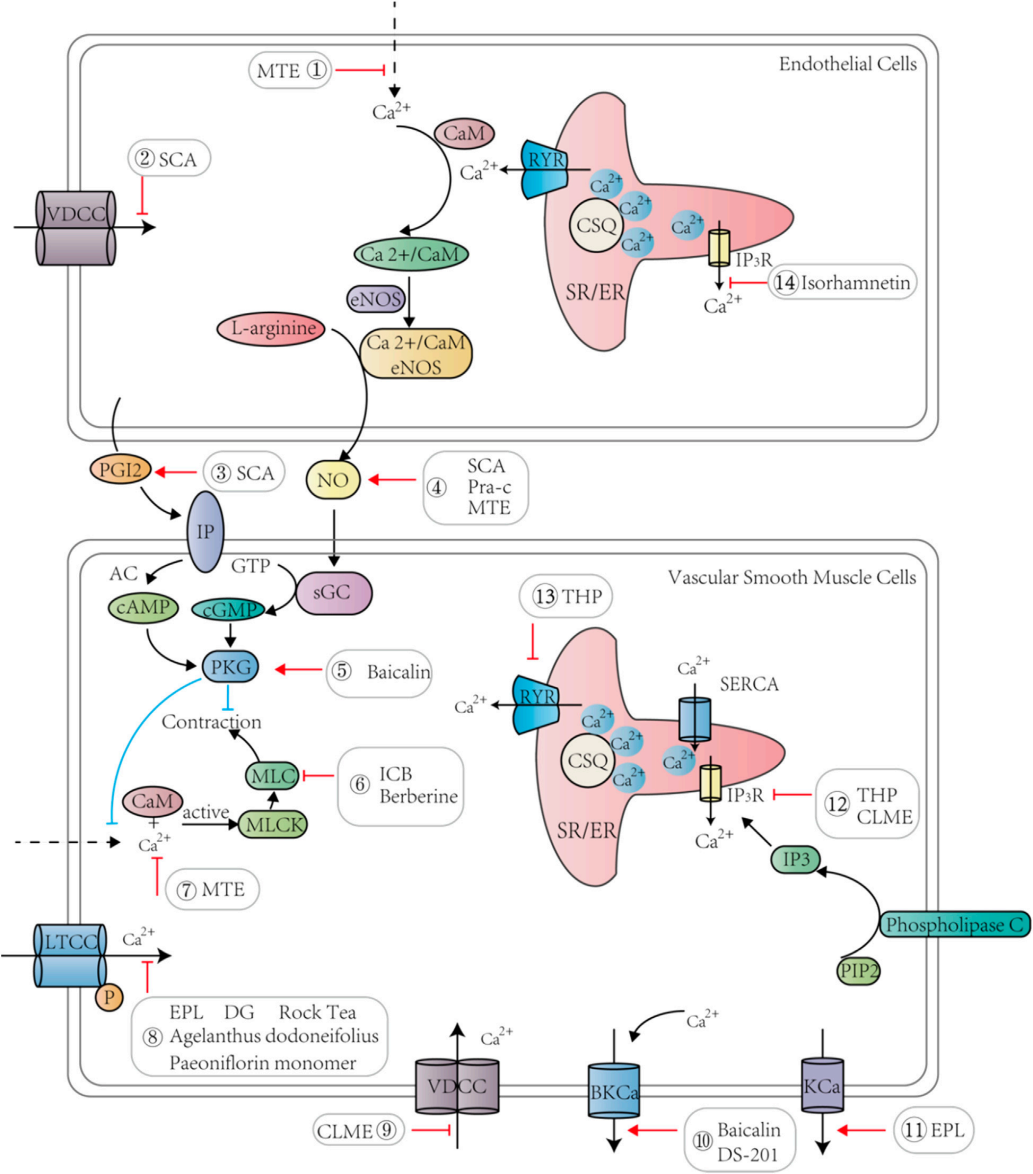
TCM and its active ingredients promote vasodilation and lower blood pressure by regulating calcium signaling pathways in endothelial cells and vascular smooth muscle cells.

Several studies have revealed that common clinical prescriptions of Chinese medicines, such as Qianhu, Schisandrae, and *Salvia miltirrhiza* (Wenjie et al., 2014; Ye et al., 2018; Hu et al., 2012), treat hypertension by regulating calcium signaling (Table 3). The hypotensive effects and related mechanisms of MTE, the water-soluble portion of *Marsdenia tenacissima,* were evaluated by the isometric vessel tension study, and the results suggested that MTE induced vasodilation by reducing calcium influx and promoting endothelial NO release (Hao et al., 2019). Bottino Pontes et al. studied Pimpinella seed aqueous extract (AE) by evaluating its effects on arterial blood pressure, and the authors found that AE could lower blood pressure and promote bradycardia. This protective cardiovascular effect was attributed to calcium entry blockage in cardiomyocytes and VSMCs (Pontes et al., 2019). *Schisandra chinensis* contains an active substance called α-Iso-cubebene (ICB), which could reduce vascular tension by inhibiting the phosphorylation of the myosin light chain (MLC) and regulate the receptor-operated channel-mediated cytosolic calcium flux induced by phenylephrine (PE) or norepinephrine (NE). Therefore, ICB can potentially be used to treat vascular hypertension (Ye et al., 2018). Praeruptorin c (Pra-c) is an active ingredient extracted from *Peucedanum praeruptorum*. Persistent hypertension promotes cardiac hypertrophy and myocardial fibrosis, and experiments in spontaneously hypertensive rats showed that Pra-c lowered blood pressure, restored calcium homeostasis, and reversed ventricular remodeling by upregulating PLN, which regulated SR Ca^2+^ uptake by complexing with sarco/endoplasmic reticulum Ca^2+^-ATPase (SERCA) (Wenjie et al., 2014; Fernández-de Gortari and Espinoza-Fonseca, 2018). Berberine, an active alkaloid found in many TCMs, dilates blood vessels and reduces blood pressure. In mice, berberine exerted its antihypertensive and antiheart failure effects by inhibiting transient receptor potential channel 4 (TRPV4) and LTCCs to reduce intracellular calcium levels and to weaken CaM/MLC activity, thereby promoting VSMC relaxation (Wang et al., 2015). Tetrahydropalmatine (THP) is a bioactive ingredient derived from *Corydalis yanhusuo*. THP has been known to ameliorate hypertension by targeting the endothelium-dependent phosphatidylinositol 3-kinase (PI3K)/Akt signaling pathway, and Ca^2+^ and K^+^ channels are also involved (Zhou et al., 2019a). Studies have been conducted to investigate the effects of Danshen and Gegen (DG) on isolated coronary arteries and their potential mechanisms in treating hypertensive heart diseases. The researchers found that danshensu and salvianolic acid B, the active compound of Danshen, reduced the calcium flux of the left anterior descending artery in rats, and lithospermic acid similarly reduced the calcium level in cardiomyocytes (Hu et al., 2012). The influx of calcium in VSMCs caused cell contraction, and DG reduced [Ca^2+^]_i_ by blocking LTCCs and partially opening the inward rectifier potassium channel (K_ir_), thereby relaxing blood vessels and relieving subsequent angina (Hu et al., 2012). Therefore, reducing [Ca^2+^]_i_ and the activity of MLC in VSMCs as well as promoting NO production in endothelial cells served as the major mechanisms of TCM in treating hypertensive heart disease (Figure 2).

**TABLE 3:** The mechanism of TCM in the treatment of hypertensive heart disease through calcium signaling.

Type of TCM	TCM	Type of study	Type of cell	Mechanism of action	References
The bioactive ingredients of TCM	α-Isocubebene	*In vitro*	VSMCs	Inhibiting calcium flux into VSMC	Ye et al. (2018)
	Aqueous extract of Pimpinella anisum L. seeds	*In vivo*	Myocardial cells/VSMCs	Inhibiting calcium influx	Pontes et al. (2019)
	Berberine	*In vitro*	VSMCs	Decreasing [Ca^2+^]_i_ levels and CaM/MLC activity	Wang et al. (2015)
	Tetrahydropalmatine	*In vitro*	VSMCs	Reducing the intracellular Ca^2+^ release induced vascular tension	Zhou et al. (2019a)
	Tanshinone ⅡA sodium sulfonate	*In vivo, in vitro*	VSMCs	Depending on the large conductance calcium-activated potassium (BK_Ca_) channels.	Zhou et al. (2019b)
	Extract of curcuma longa L.	*In vitro*	VSMCs	Blocking extracellular calcium influx and/or inhibition of intracellular Ca^2+^ release	Adaramoye et al. (2009)
Chinese Medicine decoction	Danshen and Gegen decoction	*In vitro*	VSMCs	Blocking [Ca^2+^]_i_	Hu et al. (2012)
					

### Heart Failure

Congestive heart failure is a complex disease featuring impaired heart function, during which cardiac output is insufficient to accommodate venous return and cannot provide or maintain the required basic energy or metabolism (Li et al., 2017; Smith and Eisner, 2019). In a failing heart, the Ca^2+^ transient amplitude of cardiomyocytes is decreased, together with enhanced SR Ca^2+^ leakage and reduced SR Ca^2+^ uptake (Balke and Shorofsky, 1998; Luo and Anderson, 2013). In the early stages of HF, CaMKII promotes the phosphorylation of RyR2 at serine 2814/2815, leading to abnormal Ca^2+^ signals, such as a high level of calcium sparks (Chen et al., 2020).

Many active ingredients of TCM have played an important role in anti-heart failure by inhibiting calcium signal disorder and reducing Ca^2+^ overload (Li et al., 2018a; Cheng et al., 2018; Liu et al., 2016) (Table 4). Stachydrine hydrochloride alleviated phenylephrine-induced elevated levels of sarcomere contraction, calcium transients, and calcium sparks. Moreover, among a series of proteins in the calcium signaling cascade, the hyperphosphorylation of CaMKII, RyR2, and PLN was blocked by stachydrine hydrochloride, which facilitated the binding of FKBP12.6 to RyR2 (Chen et al., 2020). Stachydrine hydrochloride also decreased the level of SR calcium leakage into the cytoplasm, and the underlying mechanism might be related to the reduction of transverse aorta constriction (TAC)/PE-induced hyperphosphorylation of CaMKII (Chen et al., 2020). An *in vivo* model of abdominal aortic constriction in rats and an *in vitro* model of ISO-treated H9C2 cells have been used to study the beneficial effect of baicalein in *Scutellaria baicalensis* on myocardial function (Zhao et al., 2016), and the results indicated that baicalein regulated the expression and activity of SERCA and RyR2 to maintain calcium balance in the heart. Additionally, baicalin inhibited the elevated expression of sodium-calcium exchanger 1 (NCX1) and phospho-CaMKII (P-CaMKII) during HF, which played an essential role in regulating [Ca^2+^]_i_, thereby protecting myocardial structure and contractile function (Zhao et al., 2016). *Sophora flavescens* contains various biologically active components with pharmacological activities, such as sophoridine (Zhao et al., 2016), which protects against HF by increasing the activity of DHPR to improve CICR (Hu et al., 2016). Astragaloside IV (AS-IV), extracted from *Astragalus membrananceus* (Huangqi), also has the potential to treat HF because it inhibits LTCCs and protects against myocardial damage caused by Ca^2+^ overload (Zhao et al., 2017). In summary, TCM modulated the expression and activities of numerous calcium handling proteins to rescue the abnormal calcium homeostasis observed in failing cardiomyocytes, such as enhanced SR calcium leakage and Ca^2+^ overload (Figure 1).

**TABLE 4:** The mechanism of TCM in the treatment of heart failure through calcium signaling.

Type of TCM	TCM	Type of study	Type of cell	Mechanism of action	References
The bioactive ingredients of TCM	Baicalein	*In vitro*	H9C2	Downregulation of phosphorylation of CaMKII and expression of NCX1, upregulation of SERCA2 and RYR2	Zhao et al. (2016)
	Stachydrine hydrochloride	*In vivo, in vitro*	Ventricular myocytes	Improving the calcium transient amplitudes, inhibiting the SR leakage	Chen et al. (2020)
	Sophoridine	*In vivo, in vitro*	Cardiomyocytes	Upregulation of DHPR and ameliorating cardiac CICR	Hu et al. (2016)
	Astragaloside IV	*In vitro*	Cardiomyocytes	Altering calcium homeostasis, inhibition of calcium influx and promotion of calcium release from SR.	Zhao et al. (2017)

### Coronary Heart Disease and Myocardial Infarction

Coronary heart disease includes a series of clinical conditions, such as myocardial infarction and arrhythmia, caused by atherosclerosis, which leads to narrowing of the coronary artery lumen and insufficient blood supply to the heart muscle (Lei et al., 2018; Liu et al., 2018). Coronary heart disease is the main cause of sudden cardiac death worldwide, accompanied by apoptosis and myocardial fibrosis, leading to HF (Yang et al., 2018a; Yang et al., 2018b).

Xin-Ke-Shu (XKS), Guanxinshutong capsule (GXSTC), modified Yiqi decoction (MYQ), and other Chinese herbal compound preparations are involved in the regulation of calcium signaling during the treatment of coronary heart disease and myocardial infarction (Yang et al., 2018a; Liu et al., 2017b; Yu et al., 2017) (Table 5). Xin-Ke-Shu tablet (XKS) is mainly composed of *Salvia miltiorrhiza*, *Radix Puerariae*, *Hawthorn*, *Panax Notoginseng* and *Radix Aucklandiae*. XKS improved LAD-induced myocardial infarction in rats, which was attributed to its effect on inhibition of the overexpression of CaMKII, thereby alleviating Ca^2+^ overload. Moreover, XKS also regulates lysophosphatidylcholine and long-chain fatty acid expression and maintains cell membrane stability (Yang et al., 2018a). GXSTC is commonly used in the clinical treatment of coronary heart disease, angina pectoris, and other CVDs. Systematic pharmacology studies revealed that GXSTC ameliorated myocardial injury of ischemia-reperfusion through twelve pathways (including the calcium signaling pathway) and eight calcium-related targets. Furthermore, the four main active ingredients (FMAI) of GXSTC (protocatechuic acid, borneol, cryptotanshinone, and eugenol) prevented Ca^2+^ overload and protected cardiomyocytes (Liu et al., 2018). Studies have been conducted to examine the effect of MYQ on cardiac function and infarct size in the coronary artery blockage-reperfusion model, and the results suggested that long-term MYQ treatment alleviated ischemia/reperfusion (I/R) injury by regulating Ca^2+^-handling proteins (increasing NCX1 and SERCA2a expression) and the signaling pathways of apoptosis and autophagy (Yu et al., 2017).

**TABLE 5:** The mechanism of TCM in the treatment of coronary heart disease and myocardial infarction through calcium signaling.

Type of TCM	TCM	Type of study	Type of cell	Mechanism of action	References
The bioactive ingredients of TCM	Four main active ingredients derived from Guanxin Shutong capsule	*In vivo, in vitro*	Cardiomyocytes	Inhibiting calcium overload	Liu et al. (2017b)
Chinese Medicine decoction	Modified Yi Qi decoction	*In vivo*	Myocardial cells	Regulation of apoptotic proteins, cytosolic calcium handling proteins	Yu et al. (2017)
Chinese patent medicine	Xin-ke-shu	*In vivo*	Myocardial cells	Inhibiting calcium overload	Yang et al. (2018a)

### Diabetic Heart Disease

Diabetes is a common metabolic disease that often causes various complications such as diabetic cardiomyopathy, which is usually due to the generation of advanced glycation end products, which directly damage myocardial cells (Poirier and Eckel, 2000; Athithan et al., 2019). Excessive endothelin 1 (ET-1) produced during diabetes further damages heart cells, resulting in weak cardiac contractility (Qi et al., 2008; Sena et al., 2013). During this process, the activity of calcium handling proteins in the SR is impaired, leading to the dysregulation of Ca^2+^ processing (Qi et al., 2008; Pereira et al., 2014).

The therapeutic effects of TCM on diabetic cardiomyopathy have also been investigated (Jiang et al., 2020; Liu et al., 2017c). In diabetes models established by streptozotocin injection, total triterpene acids (TTAs), an active chemical substance extracted from *Cornus officinalis*, played a beneficial role in diabetic cardiomyopathy by targeting calcium handling proteins (FKBP12.6, SERCA2a, and PLN) and normalizing calcium release from SR (Qi et al., 2008). Similarly, in streptozotocin-induced diabetic rats, total aralosides of aralia elata seem (TASAES) improved myocardial contractility and protected myocardial function, the possible mechanism of which was through increasing *I*_CaL_ and inhibiting connective tissue growth factor (Xi et al., 2009).

### Inflammation-Associated Heart Disease

Inflammation has been associated with numerous cardiovascular conditions, ranging from coronary heart disease to valvular heart diseases, and one of its well-known effects is promoting atherosclerosis (Wolf and Ley, 2019; Roifman et al., 2011). Thus, inflammation is considered an emerging target for the treatment of both inflammatory and commonplace heart diseases (Sethwala et al., 2021; Thackeray and Taqueti, 2020; Danesh et al., 2004). TCM’s anti-inflammatory properties may contribute to its therapeutic effects on CVDs (Hao et al., 2017; Li et al., 2019). For instance, a randomized clinical trial with a large sample size suggested the beneficial effect of QLQX in chronic HF treatment, and the possible mechanisms included the regulation of inflammation-related cytokines (Hao et al., 2017). Both total flavonoids from *Glycyrrhizae* radix et rhizoma (FGR) and total flavonoids from *Spatholobi* caulis (FSC) showed anti-inflammatory properties (Zhang et al., 2017). Total coumarins from *Peucedani* radix (CPR), as an anti-inflammatory herb, blocked the receptor-operated calcium channel (ROCC) and VDCC to inhibit calcium influx, thereby promoting vasodilation (Zhang et al., 2017).

## Discussion

**FIGURE 3:**
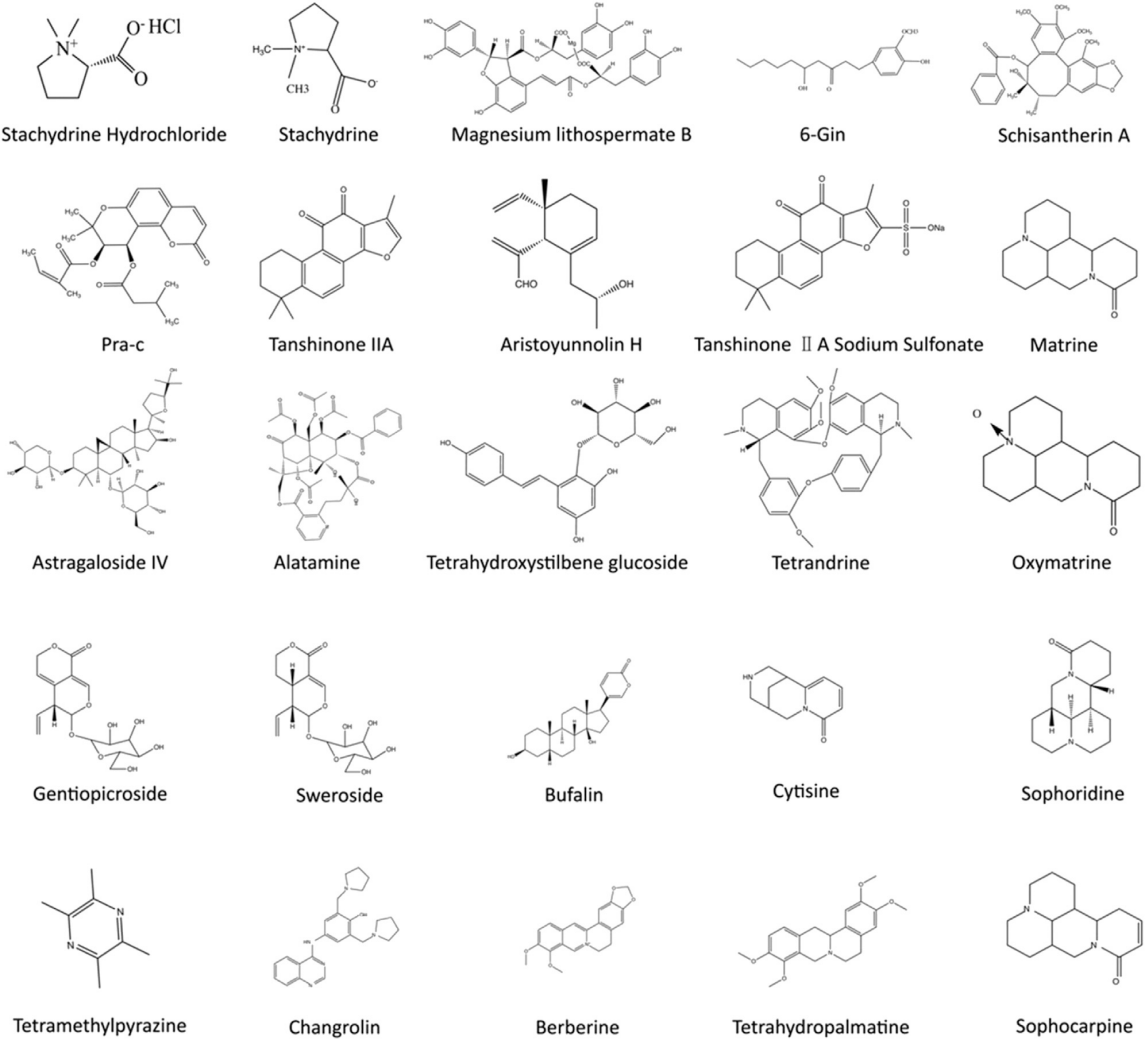
Chemical structures of TCM bioactive components that regulate calcium signaling pathways.

**FIGURE 4:**
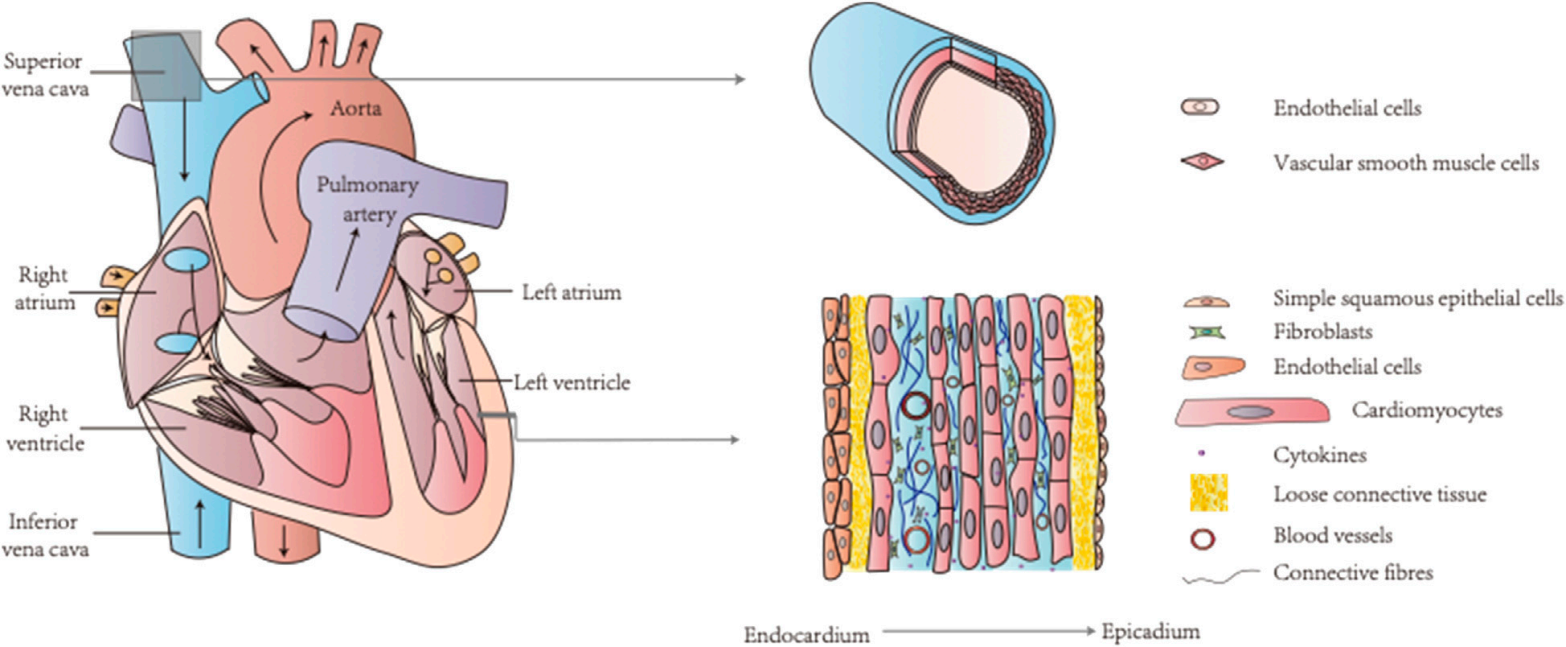
The heart and blood vessels are two key components of the circulatory system containing various types of cells. TCM can target calcium signaling in endothelial cells, vascular smooth muscle cells, and cardiomyocytes for CVDs treatment.

Overwhelming evidence suggests that TCM’s unique advantage in CVDs treatment is closely related to multiple calcium signaling pathways (Qi et al., 2008; Wang et al., 2019a; Siu and Ko, 2010). Because of the multicomponent and multitarget characteristics of TCM, diverse calcium handling proteins and signaling pathways in various types of cells involved in distinct CVDs can be regulated (Wheeler-Jones, 2005; Wang et al., 2019b; Wang et al., 2018) (Figures 3, 4). It is important to note that the structures of chemicals in TCM are highly related to their functions. For instance, the hydroxyl group in gingerol leads to a shortened aliphatic tail and decreased Van der Waals (VDW) interactions with the hydrophobic pocket of its ligand, which affects the potency of gingerol in activating Ca^2+^ permeable TRPV1 channels (Yin et al., 2019). In this review, we summarized the current research progress on the therapeutic effects of TCM on CVDs through modulation of calcium signaling.

The circulatory system includes the heart and blood vessels as two key components, and diseases of the circulatory system, i.e., CVDs, are complex and involve various cell types, including endothelial cells, VSMCs, and cardiomyocytes (Wang et al., 2019b; Li et al., 2018b; Braile et al., 2020; Rohde et al., 2015) (Figure 4). An increase in calcium levels in endothelial cells promotes NO and other endothelium-derived factors that can be released to regulate vascular tone (Hao et al., 2019). The calcium signaling pathway of endothelial cells is a common target of TCM. For example, MTE promoted vasodilation by reducing calcium influx and inducing endothelial NO release (Hao et al., 2019). Moreover, isorhamnetin inhibited IP_3_-sensitive calcium pools from releasing calcium and protected endothelial cells (Jiayi et al., 2019). Schisantherin A (SCA) regulates NO and prostacyclin (PGI2) to induce endothelium-dependent vasodilation and obstructs VDCC to promote endothelium-independent vasodilation (Yang et al., 2020b). Thus, TCM can treat certain CVD conditions such as hypertensive heart disease by maintaining proper endothelial cell function through regulation of the calcium signaling pathway (Xiong et al., 2013; Yu et al., 2013; Zhang et al., 2020) (Figure 2).

The rapid spread of cellular signals between endothelial cells and neighboring VSMCs in blood vessels is due to their tight connections (Iaizzo, 2020). NO released by endothelial cells plays a crucial role in vasodilation, mainly by activating the soluble guanosine cyclase in VSMCs and inducing the production of cGMP that further decreases [Ca^2+^]_i_ and promotes relaxation in VSMCs (Filippini et al., 2019; Hu et al., 2012; Vanhoutte et al., 2017). For vasoconstriction, VSMCs are first stimulated by extracellular signals such as vasoconstrictor agonists (Wang et al., 2018; Félétou, 2011), which results in a rapid increase and accumulation of intracellular calcium. This elevated level of calcium binds to CaM in the cytoplasm to activate downstream myosin light chain kinase (MLCK), which further phosphorylates MLC to change its conformation and promotes vasoconstriction (Hao et al., 2019; Kamm and Stull, 1985). Contraction of VSMCs regulates blood vessel tension and blood flow distribution and is closely related to atherosclerosis and coronary heart disease because of the phenotypic plasticity of VSMCs (Gomez and Owens, 2012). Some TCMs can regulate the function of VSMCs by acting on LTCCs and other calcium channels to reduce calcium levels (Figure 2). For instance, ICB promoted VSMC relaxation by blocking calcium entry and MLC phosphorylation (Ye et al., 2018). Berberine inhibited TRPV4 to reduce calcium influx and to decrease CaM/MLC activity, thereby regulating vascular tightness (Wang et al., 2015). The mechanism of tanshinone IIA sodium sulfonate (DS-201) vasodilation might be related to the activation of large-conductance calcium-dependent potassium channels (BK_ca_), making DS-201 a combination therapy candidate with a BK Ca^2+^ agonist to treat hypertension (Zhou et al., 2019b). Baicalin has also been shown to activate the BK_ca_ current, cGMP-dependent protein kinase (PKG), and PKA pathways, which increase potassium outflow and promote hyperpolarization of the myocardial cell membrane, resulting in vasodilation (Lin et al., 2010). EPL promoted vasodilation by activating calcium-activated potassium channels (K_Ca_) and ATP-sensitive K^+^ channel (K_ATP_) channels and suppressing LTCCs to enhance the Akt- and NO-cGMP signaling pathways (Jin et al., 2012). Certain TCMs can also act on intracellular Ca^2+^ release to facilitate VSMC relaxation. For instance, the methanolic extract of Curcuma longa (CLME) prevented calcium release from the IP_3_-sensitive calcium store to promote vasodilation (Adaramoye et al., 2009). Therefore, TCM directly targets the calcium signaling pathway in VSMCs to promote vasodilation.

The heart is responsible for pumping blood to provide nutrition for various organs and tissues, which depends on the cardiomyocytes' rhythmic contraction and relaxation (Woodcock and Matkovich, 2005). After being excited, the membrane of cardiomyocytes will have action potentials caused by sequential opening and closure of multiple ion channels (Balse et al., 2012). In this process, activation of voltage-gated LTCCs promotes Ca^2+^ influx from the external environment, and the elevated calcium level in the cytoplasm activates SR to release more calcium ions (Woodcock and Matkovich, 2005; Zhu et al., 2019). Ca^2+^ then binds to troponin to induce cardiac contraction (Park et al., 2017). Thus, the proper regulation of intracellular calcium ions is crucial for maintaining heart functions (Marks, 2003). Indeed, the disturbance of calcium homeostasis has been observed in numerous types of CVDs, such as cardiac arrhythmias (Landstrom et al., 2017). Various studies have shown that Chinese herbal medicines and their active ingredients can improve cardiomyocyte function and treat CVDs by regulating the expression and activity of calcium channels and receptors (Hu et al., 2012; Yang et al., 2020b; Liu et al., 2013) (Figure 1). For example, senkyunolide A and ligustrazinecan hindered the opening of VDCC, rendering a calcium antagonistic effect (Lei et al., 2018). Magnesium lithospermate B (MLB) could be used to treat angina pectoris and coronary heart disease by reversibly inhibiting L-type Ca^2+^ currents (Wang et al., 2006). In addition to regulating LTCCs, which are responsible for the Ca^2+^ influx in cardiomyocytes, RyRs that mediate intracellular Ca^2+^ release from SR are also valid targets for TCM (Lei et al., 2018). FMAI from Guanxin Shutong capsule increased the expression of RyR2 and PLN in cardiomyocytes (Liu et al., 2017b). As a crucial calcium removal mechanism, NCX, in its forward mode, pumps 1 Ca^2+^ out of cells in exchange for 3 Na^+^ to reduce [Ca^2+^]_i_ (Pott et al., 2011). *Ginkgo biloba* extract 50 (GBE50) reduced NCX abnormal expression to improve myocardial contractility (Liu et al., 2013). On the other hand, ginseng–aconite decoction (GAD) exerted a positive inotropic effect on cardiomyocytes by activating NCX’s reverse mode to induce Ca^2+^ entry (Cui et al., 2013). As a result of modulating multiple calcium handling proteins, TCM’s common effect is preventing Ca^2+^ overload, which is detrimental for cardiomyocytes (Vassalle and Lin, 2004). For example, the combination of ginsenoside Rb1, ruscogenin, and schisandrin, three representative ingredients in Sheng-Mai-San, inhibited Ca^2+^ overload and maintained cardiac histological characteristics during myocardial ischemic injury (Li et al., 2018a). Ophiopogonin reduced myocardial cell apoptosis induced by intracellular Ca^2+^ overload and endoplasmic reticulum stress (Yang et al., 2017). TCM can also regulate calcium-related signaling pathways. Ginsenoside reduced Ca^2+^ overload and altered the calcineurin signaling pathway, which improved the metabolism of ischemic myocardial tissue and protected myocardial structure (Yang et al., 2017). FMAI also reduced [Ca^2+^]_i_ and prevented apoptosis by modulating the Ca^2+^/CaM/CaMK signaling pathway and changing gene expression in cardiomyocytes (Liu et al., 2017b). In summary, accumulating evidence suggests that TCM plays an important role in CVD treatments by reducing apoptosis, protecting cardiac structure and enhancing the contractile function of cardiomyocytes through modulation of Ca^2+^ homeostasis.

## Conclusion

The pathophysiology of CVDs is multifactorial and complex, involving calcium signaling as a key player. With multicomponent and multitarget characteristics, Chinese herbal medicine has shown unique advantages in CVD treatment with few side effects. Accumulating evidence suggests that TCM plays an important role in maintaining calcium homeostasis and improving calcium signaling to alleviate the symptoms of CVDs. Thus, we reviewed the positive effects of TCM and its active ingredients on a wide range of CVDs and highlighted the central role of calcium signaling in their mechanism of action in endothelial cells, VSMCs and cardiomyocytes, which may provide basic guidance for follow-up research.

## Glossary

AF: atrial fibrillation
Ang II: angiotensin II
ANP: atrial natriuretic peptide
APS: astragalus polysaccharide
AS-IV: astragaloside IV
BNP: brain natriuretic peptide
CaM: calmodulin
CaMKII: calmodulin-dependent protein kinase II
cAMP: cyclic adenosine monophosphate
cGMP: cyclic guanosine monophosphate;
CICR: calcium-induced calcium release
CLME: methanolic extract of curcuma longa
CPR: total coumarins from Peucedani radix
CSQ: calsequestrin
CVDs: cardiovascular diseases
DeHE: dehydroevodiamine alkaloid
DG: danshen and gegen decoction
DHBF: dracocephalum heterophyllum benth flavonoid
DHPR: dihydropyridine receptor
DS-201: tanshinone IIA sodium sulfonate
eNOS: endothelial nitric oxide synthase
EPL: ethanol extract of paeonia lactiflora pall
ET-1: endothelin 1
FMAI: four main active ingredients
FSC: total flavonoids from spatholobi caulis
GAD: ginseng–aconite decoction
GBE50: ginkgo biloba extract 50
GXSTC: guanxinshutong capsule
HF: congestive heart failure
I/R: ischemia/reperfusion
ICa: calcium currents
ICaL: L-type calcium current
ICB: α-Iso-cubebene
IK: delayed rectifier current
IK1: inward rectifier potassium currents
INa: sodium current
INaL: the late sodium curren
INCX: sodium-calcium exchanger current
IP3R: Inositol trisphosphate receptor
ISO: isoprenaline
Ito: transient outward potassium current
JP2: junctophilin-2
KATP channel: ATP-sensitive K+ channel
KCa channel: calcium-activated potassium channel;
Kir channel: inward rectifier potassium channel
LTCC: L-type calcium channel
MLB: magnesium lithospermate B
MLC: myosin light chain
MLCK: myosin light chain kinases
MTE: marsdenia tenacissima extract
MYQ: the modified yiqi decoction
NCX: sodium-calcium exchanger
NE: norepinephrine
NFAT: nuclear factor of activated T cells
NO: nitric oxide
P-CaMKII: Phospho-CaMKII
PE: phenylephrine
PGI2: prostacyclin
PI3K: phosphatidylinositol 3-kinase
PKA: cAMP-dependent protein kinase
PKG: cGMP-dependent protein kinase;
PLN: phospholamban
Pra-c: praeruptorin c
QLQX: qiliqiangxin
RCT: randomized controlled trial
RyR: ryanodine receptor
SCA: schisantherin A;
SERCA: sarco/endoplasmic reticulum Ca2+-ATPase
sGC: soluble guanylate cyclase
SR: sarcoplasmic reticulum
STA: stachydrine
TAC: transverse aorta constriction
TASAES: total aralosides of aralia elata seem
TCM: traditional Chinese medicine
TFAS: total flavones from acanthopanax senticosusa
TFLF: tu-fu-ling flavonoids;
THP: tetrahydropalmatine
TRPV4: transient receptor potential channel 4
TTAs: total triterpene acids
VDCC: voltage-dependent calcium channel
VSMCs: vascular smooth muscle cells
WXKL: wenxin keli
XKS: xin-ke-shu
β-AR: β-adrenergic receptors
β-MHC: β-myosin heavy chain
